# Effects of Different Concentrate Feed Proportions on Ruminal Ph Parameters, Duodenal Nutrient Flows and Efficiency of Microbial Crude Protein Synthesis in Dairy Cows During Early Lactation

**DOI:** 10.3390/ani10020267

**Published:** 2020-02-08

**Authors:** Katharina Bünemann, Maren Johannes, Rolf Schmitz, Julia Hartwiger, Dirk von Soosten, Liane Hüther, Ulrich Meyer, Heiner Westendarp, Jürgen Hummel, Annette Zeyner, Sven Dänicke

**Affiliations:** 1 Institute of Animal Nutrition, Friedrich-Loeffler-Institut (FLI), Federal Research Institute for Animal Health, 38116 Braunschweig, Germany; Katharina.Buenemann@fli.de (K.B.); maren.johannes@gmail.com (M.J.); rolf_schmitz@gmx.net (R.S.); juliahartwiger@gmx.de (J.H.); Liane.Huether@fli.de (L.H.); Ulrich.Meyer@fli.de (U.M.); Sven.Daenicke@fli.de (S.D.); 2 Faculty of Agricultural Sciences and Landscape Architecture, University of Applied Sciences, 49076 Osnabrück, Germany; h.westendarp@hs-osnabrueck.de; 3 Department of Animal Sciences, University of Göttingen, 37077 Göttingen, Germany; jhummel@gwdg.de; 4 Institute of Agricultural and Nutritional Sciences, Martin-Luther-University Halle-Wittenberg, 06120 Halle (Saale), Germany; annette.zeyner@landw.uni-halle.de

**Keywords:** dairy cows, concentrate feed proportion, ruminal pH, nutrient flows, microbial efficiency, postpartal period

## Abstract

**Simple Summary:**

Around calving, cows exhibit a depression in feed intake. An imbalance between energy intake and energy demands occurs, which results in a negative energy balance. Concentrate feed proportions of the ration are increased to compensate that energy deficit. The accompanying increase in concentrate intake leads to higher production of short chain fatty acids, which in turn might lower the ruminal pH. A ruminal pH < 5.8 for a certain period of time can lead to subacute ruminal acidosis. Keeping the ruminal pH within the physiological range is important for microorganisms colonizing the rumen. Those microorganisms metabolize feed protein via ammonia or amino acids to microbial protein, which is then available for the host. Microbial efficiency is limited by availability and balance of nitrogen and energy, the latter is mostly provided as starch. The aim of the present study was to examine influences of different concentrate feed proportions and of microbial efficiencies on ruminal pH parameters, nutrient flows and digestibilities. Therefore, cows were additionally grouped according to their individual microbial efficiency. The concentrate treatment effect did not cause differences in the mentioned parameters. However, more microbial efficiency cows exhibited higher nutrient flows but lower digestibilities.

**Abstract:**

The aim of the study was to examine different pH parameters, such as variations throughout the day, depending on differing concentrate feed proportions. Moreover, special attention was payed to individual variation in microbial efficiencies (microbial crude protein/fermented organic matter) and their relation to ruminal pH, nutrient flows and digestibilities. For this, cows were grouped according to microbial efficiency (more, n = 5, vs. less efficient cows, n = 4). After calving, thirteen ruminally cannulated pluriparous cows, including nine duodenally cannulated animals, were divided into groups offered rations with a lower (35% on dry matter basis, n = 7) or a higher (60% on dry matter basis, n = 6) concentrate feed proportion. Ruminal pH parameters were assessed continuously by using intraruminal probes. Nutrient flows, nutrient digestibility and microbial efficiency were determined for duodenally cannulated cows. For most ruminal pH parameters it seemed that individual variability was higher than the treatment effect. However, a positive relationship between actual concentrate intake and diurnal pH fluctuations was found. Besides, the effect of individually different microbial efficiencies was assessed. Again, there were no group differences for pH parameters. However, nutrient flows were significantly higher in more efficient cows, whereas digestibilities were lower in in more efficient cows.

## 1. Introduction

Cows display a depression in feed intake during the transition period, which leads to an imbalance between energy intake and energy demands [1,2]. To balance this discrepancy, concentrate feed proportions of the rations (C) are often elevated post partum (p.p.). The increase of concentrate intake is accompanied by an increased production of short chain fatty acids (SCFA) in the rumen, which can affect the ruminal pH negatively [3,4]. To keep the ruminal pH within the physiological range, the absorption of SCFA through the ruminal epithelium has to increase simultaneously [5,6]. Another factor to compensate the elevated SCFA concentration is an increased chewing activity, which in turn increases saliva production and buffer secretion. Disappearance of SCFA is also determined by passage rate to the lower digestive tract [7]. If the imbalance between production and elimination of SCFA impacts the ruminal pH in a way that it drops below an appropriate threshold for a certain period of time, this can result in subacute ruminal acidosis (SARA) [7,8,9]. The risk of developing SARA is particularly high during the transition period, with a shift from a dry period to an early lactation diet [10]. Detection of SARA is difficult. Nevertheless, for the attempt of definition, thresholds according to Zebeli et al. [9] are used most frequently. These are described as a daily mean pH < 6.16 and the time per day with pH < 5.8 for more than 5.24 hours. However, several studies assume high individual variability in ruminal pH and in susceptibility to develop SARA [1,11,12].

A low ruminal pH can also reduce fiber digestion and therefore lower microbial efficiency, which is defined as synthesized microbial protein per fermented organic matter (mCP/fOM) [13,14,15]. A decrease of fiber digestion can impede an even energy provision throughout the day, which is necessary for an optimal microbial growth. The release of energy from non-structural carbohydrates is faster than the energy consumption of the microorganisms and can result in an uncoupled fermentation. This again can lead to energy spilling [16,17]. Energy is used for the so-called non-growth processes, such as maintaining the intracellular pH on an optimal level instead of using it for cell growth under low pH conditions [16,18]. However, most studies assumed stable ruminal pH conditions while variations throughout the day received little attention [19]. Microbial efficiency depends on availability and balance of nitrogen (N) and fermentable energy [15,18,20]. Fermentability of the diet might in turn influence not only the ruminal pH, but also the passage rate [18]. The fermentability of the diet is primarily influenced by fermentability of the contained starch. Starch fermentation is accompanied with an increase of SCFA concentration, which lowers ruminal pH. In addition, a high amount of starch can increase passage rate, due to a smaller particle size of feedstuffs with higher starch contents compared to forage [5,18,21]. Yet, few studies have been conducted on the relationship between ruminal pH, nutrient flows and microbial efficiency. Firkins et al. [22] demonstrated an improvement of 39% in microbial efficiency, due to a 15% decrease of organic matter digestibility. Therefore, they considered the increasing passage rate to be a general explanation. They proposed that substrate supply increases with enhanced passage rate, which would improve cell growth, due to an increase of growth related enzymes in the microorganisms. Additionally, an increased passage rate can decrease microbial turnover in the rumen [23]. However, investigations differ in their results concerning this relation, as higher passage rates can also lead to microbial washout [24,25].

The objective of the present study was, on the one hand, to examine different ruminal pH parameters depending on differing concentrate feed proportions, thereby, paying special attention to diurnal fluctuations. Besides, the concentrate effect on microbial efficiency, nutrient flows and digestibility was assessed. On the other hand, the study intended to investigate the relation between ruminal pH and microbial efficiency from a new perspective for gaining further information. Therefore, the microbial efficiency was chosen as starting point, by grouping the cows according to their individual microbial efficiency (more vs. less efficient). For this, the average microbial efficiency of 156 g mCP/kg fOM according to GfE [15] was used as threshold. Moreover, the effect of microbial efficiency on nutrient flows and digestibilities was examined. For this reason, different statistical evaluations were applied in order to appraise both the effects of varying dietary energy concentration as well as those of microbial efficiency on the parameters mentioned.

## 2. Materials and Methods

The experiment was performed in compliance with the German legislation on animal protection (Animal Welfare Act) and approved by the Lower Saxony State Office for Consumer Protection and Food Safety (LAVES, Oldenburg, Germany) in consultation with an independent ethics committee (AZ 33.19-42502-04-15/1858).

### 2.1. Experimental Design

Two diet types were created in order to induce distinct differences in energy supply and presumably ruminal pH parameters. While the high-caloric diet was designed to contain 60% concentrate feed, the low-caloric ration was contrasted with 35% concentrate feed.

The current study was based on 13 ruminally cannulated, pluriparous German Holstein cows, including nine additionally duodenally cannulated animals. Rumen cannula enables the access to the reticulo-rumen via the dorsal sac of the rumen. The duodenal cannula was inserted in the proximal duodenum. The experiment covered the period from 3 weeks before calving until 70 days in milk (DIM) and one additional week of duodenal chyme sampling (cows were on average in the 13th week of lactation ± 16 days). Before parturition, all cows received the same standardized total mixed ration (TMR) consisting of 80% silage (70% maize silage, 30% grass silage on dry matter (DM) basis) and 20% concentrate on DM basis. After calving, animals were divided into two groups and assigned to two different concentrate feed proportions. Cows received a partial mixed ration (PMR) consisting of 48% maize silage, 20% grass silage and 32% concentrate feed. Rations for the groups with lower concentrate feed proportion (C_35_) were adjusted to 35% concentrate feed by using automatic feeding stations (Intensec, B.V., Marknesse, The Netherlands). For the groups with a higher amount of concentrate (C_60_), C was also provided by the automatic feeders and increased from 35% to 60% during the first three weeks p.p. The components and the chemical compositions of the feedstuffs are presented in Table 1 and Table 2.

### 2.2. Measurements and Sample Collections

#### 2.2.1. Dry Matter Intake and Milk Yield

Dry matter intake (DMI) was recorded for both PMR and concentrate individually by computerized feeding stations (Insentec, B.V., Marknesse, The Netherlands). Milking took place at 05:30 a.m. and 03:30 p.m. and milk yield was determined by automatic milk counters throughout the 70 DIM (Lemmer Fullwood GmbH, Lohmar, Germany).

#### 2.2.2. Feed and Milk Samples

Samples of the mixed ration components were taken twice a week and pooled to a collective sample every four weeks. Samples of concentrate were collected once a week and also pooled to a collective sample monthly. Milk samples were taken twice a week during morning and evening milking and stored at 4 °C until analysis.

#### 2.2.3. Rumen Fluid Samples

Rumen fluid samples were taken after morning milking at eight time points, after calving (days p.p.: 3, 7, 14, 21, 28, 42, 56, 70) as well as in the week of duodenal chyme sampling. Rumen fluid samples were collected through the rumen cannula using a manual pump. With every sample, about 200 mL of rumen fluid were taken. Samples were stored at 4 °C until further analysis.

#### 2.2.4. Rumen pH and Rumination Behavior Measurements

A ruminal pH measuring device (Lethbridge Research Centre Ruminal pH Measurement System, Dascor, Escondido, CA, USA) was used to record the pH values in the ventral sac of the rumen continuously. The pH values were recorded every minute and measured during several consecutive 24-h periods each week (2 ± 1.16; mean ± SD) from week -3 to week 10 relative to calving, as well as in the week of duodenal chyme sampling. At some periods data of individual cows were missing, due to either insufficient capacity of instruments or technical problems. However, it was ensured that at least three cows of every group were recorded in every period. Before and after each measuring period the system was calibrated in buffer solutions of pH 4 and 7 at 39 °C.

#### 2.2.5. Duodenal Chyme and Faeces Samples

Each duodenally cannulated cow received a chromium oxide marker (Cr_2_O_3_) for 16 consecutive days. Marker administration started when cows were 73 DIM (± 16) on average. During the first 10 days, the marker was inserted into the rumen through the rumen cannula in two portions of 50 g each at 05:00 a.m. and 5:00 p.m. During the last 6 days, the marker was inserted in four portions of 25 g every 6 h. The chromium oxide marker dosing schedule was performed according to Schäfers et al. [26]. During the last 5 days of the marker administration period, samples of duodenal chyme were collected in intervals of 10 h. Samples were collected from the duodenal cannula and pooled to a collective sample for the five sampling days and stored at −20 °C until further analysis.

Faeces samples were taken rectally with every duodenal chyme sampling and then pooled to a collective sample.

### 2.3. Analyses

#### 2.3.1. Feed and Milk Analyses

Feed samples were analyzed according to the standard methods of the Association of German Agricultural Analysis and Research Centers (VDLUFA, method numbers are given hereafter) [15]. PMR components and concentrate were analyzed for DM (3.1), crude ash (8.1), crude protein (Dumas method, 4.1.2), starch (7.2.1, polarimetric method), ether extract (5.1.1), neutral detergent fiber (aNDF_om_, 6.5.1) and acid detergent fiber (ADF_om_, 6.5.2) (Table 2).

Milk samples of 20 morning and 20 evening samples were analyzed for fat, protein, and lactose by an infrared milk analyzer (Milkoscan FT 6000; Foss Electric, Hillerød, Denmark).

#### 2.3.2. Rumen Fluid Analyses

Short chain fatty acids (SCFA) were determined according to Geissler et al. [27]. For this, rumen fluid samples were centrifuged (5× 2400 *g*) (Beckman J2-H2, Beckman Coulter Inc., Brea, CA, USA), 10 mL of the fluid phase were added to 1 mL of 25% sulphuric acid and centrifuged again (20× 2700 *g*) (Eppendorf 5417 R, Eppendorf AG, Hamburg, Germany). The supernatant was filled into GC-vials. Afterwards it was separated by gas chromatography (Clarus 680 CG, Perkin Elmer, Waltham, MA, USA) with a polyethylene glycol capillary column (Zebron ZB-FFAP, 30 m × 0.32 mm i.d., 0.5 µm film thickness, Phenomenex LTD, Aschaffenburg, Germany) and a flame ionization detector.

Ammonia-N was analyzed using a steam distillation according to DIN38406-E5–2 within two hours after fluid sampling [28].

#### 2.3.3. Duodenal Chyme and Faeces Analyses

After thawing, 2 × 60 mL of each duodenal chyme sample were filled in 100 mL Kautex bottles. To ensure a representative proportion of solid and fluid components, the sub-sampling was done under constant stirring. The remainders of the duodenal chyme samples, as well as the pooled faeces samples were freeze dried (CHRIST, Osterode am Harz, Germany). Analysis of DM of duodenal chyme and faeces samples was performed according to VDLUFA method 3.1 [29]. Faeces samples were also analyzed for aNDF_om_ and ADF_om_. Furthermore, the 60 mL samples of duodenal chyme were used to determine the total nitrogen content according to Kjeldahl method (VDLUFA method 4.1.1) and the ammonia content according to DIN 38406-E5-2 [28,29]. The freeze dried samples of duodenal chyme and faeces were, besides, used to analyze the chromium concentration. For this, samples were prepared according to Williams et al. [30]. Chromium content of the freeze dried duodenal chyme and faeces samples was determined using an optical emission spectrometer with inductively coupled plasma (ICP-OES Quantima; GBC Scientific Equipment Pty. Ltd., Melbourne, VIC, Australia). The proportion of microbial nitrogen in duodenal chyme was determined at a wavelength of 800-2400 nm by using a NIR spectroscopy according to Lebzien and Paul [31].

### 2.4. Calculations

Computation of daily duodenal dry matter flow (DMF) was based on the equation according to Pappritz et al. [32]:

(1) DMF (kgday)= (application of Cr2O3 in mgcowdayCr2O3 in duodenal chyme (mggDM))/1000

The following formulas were used to calculate non-ammonia-nitrogen (NAN) proportion at the duodenum:

(2) $$\mathrm{Total}\;N\;\mathrm{in}\;\mathrm{duodenal}\;\mathrm{chyme}\;\left(\%\;\mathrm{of}\;\mathrm{DM}\right)=\frac{\mathrm{Total}\;N\;\left(\%\;\mathrm{of}\;\mathrm{fresh}\;\mathrm{matter}\;\left(\mathrm{FM}\right)\right)}{\mathrm{DM}\;\mathrm{of}\;\mathrm{duodenal}\;\mathrm{chyme}\;\left(\%\right)}\times 100$$

(3) $${\mathrm{NH}}_{3}N\;\mathrm{in}\;\mathrm{duodenal}\;\mathrm{chmye}\;\left(\%\;\mathrm{of}\;\mathrm{DM}\right)=\;\frac{{\mathrm{NH}}_{3}N\;\mathrm{in}\;\mathrm{duodenal}\;\mathrm{chyme}\;\left(\%\;\mathrm{of}\;\mathrm{FM}\right)}{\mathrm{DM}\;\mathrm{of}\;\mathrm{duodenal}\;\mathrm{chyme}\;\left(\%\right)}\times 100$$

(4) $$\mathrm{NAN}\;\mathrm{proportion}\;\mathrm{in}\;\mathrm{duodenal}\;\mathrm{chyme}\;(\%\;\mathrm{of}\;\mathrm{DM})\;=\;\mathrm{Total}\;N\;\mathrm{in}\;\mathrm{duodenal}\;\mathrm{chyme}\;(\%\;\;\mathrm{of}\;\mathrm{DM})\;-{\;\mathrm{NH}}_{3}N\;\mathrm{in}\;\mathrm{duodenal}\;\mathrm{chyme}\;(\%\;\mathrm{of}\;\mathrm{DM})$$

Duodenal NAN flow (kg/day) was calculated by multiplying the NAN proportion in duodenal chyme (% of DM) by DMF (kg/day).

The microbial crude protein (mCP) was calculated using the following formula:

mCP (g/day) = [duodenal NAN flow (kg/day) × (microbial N proportion of NAN (%))/100] × 6.25(5)

According to GfE [15] and Pappritz et al. [32] microbial organic matter (mOM), and ruminal fOM were calculated as follows:

(6) mOM (kg/day)=11.8×microbial N (kg/day)

fOM (kg/day) = OM intake (kg/day) − [duodenal OM flow (kg/day) − microbial OM (kg/day)](7)

Microbial efficiency was calculated as mCP per fOM according to GfE [15].

Digestibility quotient of aNDF_om_ and ADF_om_ was calculated according to Simon [33] for the total digestive tract and at the duodenum. An example for the calculation is given for digestibility of aNDF_om_ at the duodenum.

digestibility quotient of aNDFom (%) = [(aNDFom intake (kg/day) − aNDFom at the duodenum(kg/day))/aNDFom intake (kg/day)] × 100(8)

The equation according to McGinn et al. [34] was used to calculate total digestive tract digestibility of DM (tdDM):

(9) $$\mathrm{tdDM}\;(\%)\;=\;1\;-\;[({\mathrm{Cr}}_{2}O_{3}\;\mathrm{in}\;\mathrm{marker}\;(\mathrm{mg}/\mathrm{day}))/\mathrm{DM}\;\mathrm{intake}\;(\mathrm{kg}/\mathrm{day})]/{\;\mathrm{Cr}}_{2}O_{3}\;\mathrm{in}\;\mathrm{faeces}\;\;\times \;(\mathrm{mg}/\mathrm{kg}\;\times \;\mathrm{DM})$$

### 2.5. Statistical Analysis

For ruminal pH data were summarized to means of every measuring period for each cow and hence weekly means were calculated.

Weekly means were also calculated for performance parameters, milk parameters and proportion of SCFA and ammonia concentration in rumen fluid. For statistical analyses, two weeks were merged to one period for every parameter, which resulted in five periods.

For statistical analyses of duodenal chyme samples, mean values for the five sampling days were calculated for each cow and each parameter.

The statistical evaluation with the SAS software package (version 9.4.; SAS Institute Inc., Cary, NC, USA) included the data collected after parturition when cows received different diets. Performance parameters, proportion of SCFA and ammonia concentration values in rumen fluid, as well as rumen pH parameters were analyzed by using the MIXED procedure for repeated measures with a compound symmetry structure [35]. C and period were applied as fixed effects, as well as the interaction between them. Each cow within treatment was considered to be a random effect. The period of sampling was treated as a repeated measure.

**Figure 1:**
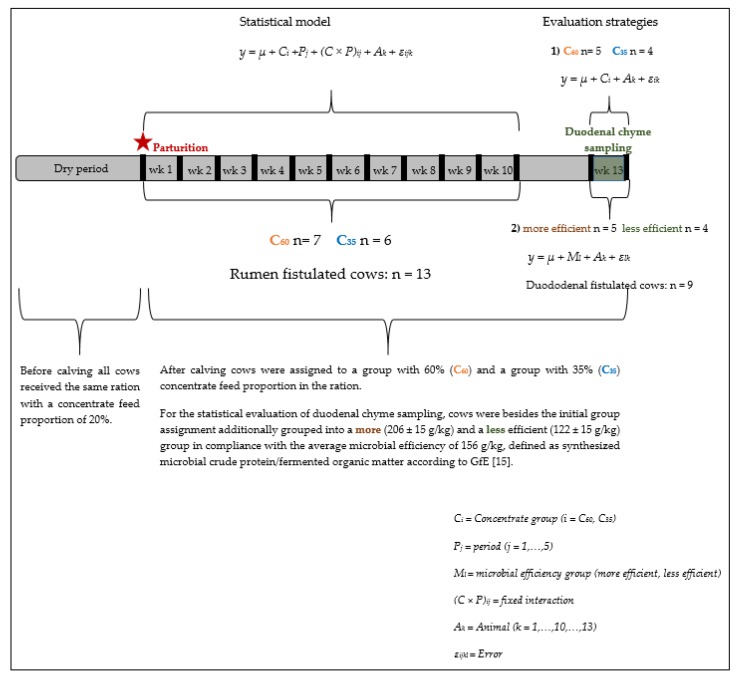
Experimental setup and statistical evaluations.

For the analysis of the duodenal chyme samples with regard to the estimated parameters, we wanted to assess both the effect of rations differing in C, as well as individually different microbial efficiencies (defined as mCP/fOM, according to GfE [15]) concerning nutrients flows, digestibilities, and ruminal pH. As duodenal chyme was sampled in only one period, the statistical evaluation included just a simple t-test with two different grouping strategies. First, concentrate feed proportion (C_60_ vs. C_35_) was used for grouping. Secondly, cows were grouped according to microbial efficiency (more and less efficient, see Figure 1). For this, the mean microbial efficiency of 156 g mCP/kg fOM according to GfE [15] was used as threshold. Cows with an individual microbial efficiency < 156 g/kg were considered to be less efficient, whereas cows with an individual microbial efficiency > 156 g/kg were considered to be more efficient. The individual microbial efficiencies for the week of duodenal chyme sampling are presented in Table A2. The statistical evaluations with either C or microbial efficiency as fixed factors are presented in Figure 1.

By assessing Pearson’s correlation coefficient, we examined relations between parameters applying the statistical software TIBCO Statistica (Version 13.3, TIBCO Software Inc., Palo Alto, CA, USA) for both approaches. Furthermore, we performed linear regression analysis, if suitable.

The MIXED procedure, the t-Test and the correlation coefficient (r) were considered statistically significant when *p* ≤ 0.05 and highly significant when *p* ≤ 0.01 while a trend was assumed for 0.05 < *p* < 0.1.

In the following, results are presented as LSMean ± standard error of means (SEM) for the MIXED procedure, and as means ± standard deviation of means (SD) for the t-Test, unless otherwise stated.

To analyze the diurnal ruminal pH variation, the values β0 and β1 were estimated by using PROC NLMIXED in SAS 9.4 (version 9.4; SAS Institute Inc., Cary, NC, USA). A logistic curve was fitted for every 24 hour interval, whereby β0 illustrates the slope of the regression line at the inflection point and therefore displays the variation of rumen pH over 24 h (the greater the values the more constant the ruminal pH) while β1 represents the inflection point of the curve and is an indicator for the average pH of the 24 h period [36]. The logistic curve depends on three parameters, namely the slope (β0) of the upper limit (β2) and the inflection point (β1) of the curve. The following formula describes this association:

(10) $$y=\;\frac{\beta 2}{1+\exp\;\times \;\left[-\beta 0\;\times \left(x-\;\beta 1\right)\right]}$$

The accumulated time (min/day) spent below each corresponding pH point on the x-axis, is represented on the y-axis. The ruminal pH values are represented on the x-axis. The upper limit of the curve was kept constant at 1,440 min/day. Therefore, the logistic curve is only described by β0 and β1 Colman et al. [37].

The time per day with pH <5.8 (min/day) was evaluated as described in Colman et al. [37]. Thresholds of 5.24 hours/day at pH <5.8 and a daily average pH <6.16 were set for a higher SARA risk according to Zebeli et al. (2008). For assessing the SARA risk, a SARA_risk_-Score was calculated for which the following equation according to Schären et al. [38] was used.

(11) $${\mathrm{SARA}}_{\mathrm{risk}}-\mathrm{Score}=\frac{\sum \left(\frac{\mathrm{Number}\;\mathrm{of}\;\mathrm{positive}\;\mathrm{observations}\;\mathrm{per}\;\mathrm{cow}\;\mathrm{in}\;\mathrm{period}\;i}{\mathrm{Total}\;\mathrm{number}\;\mathrm{of}\;\mathrm{observations}\;\mathrm{per}\;\mathrm{cow}\;\mathrm{in}\;\mathrm{period}\;i}\right)}{\mathrm{Total}\;\mathrm{number}\;\mathrm{of}\;\mathrm{cows}\;\mathrm{assessed}\;\mathrm{in}\;\mathrm{period}\;i}$$

For the intergroup comparison, weekly means of each parameter were calculated.

## 3. Results

### 3.1. Performance Parameters

DMI increased over time irrespective of concentrate feed proportion of the ration (*p*_period_ < 0.001, Table A1). Net energy intake, as well as starch intake increased over time more pronounced in group C_60_ compared to C_35_. (*p*_C×period_ net energy intake = 0.016, Table A1, *p*_C×period_ starch intake < 0.001, Table A1).

Milk yield increased over time in both groups, with a steeper rise in group C_35_, but an approximation of group C_60_ to group C_35_ during the last part of the trial (*p*_C×period_ < 0.001). Energy corrected milk tended to be higher for the C_35_ group (*p_C_* = 0.093) and enhanced within each group over the trial (*p*_period_ < 0.001). Milk fat content enhanced in both groups, with a more pronounced increase in group C_35_ (*p*_C×period_ < 0.001). The same is true for milk fat yield (*p*_C×period_ = 0.042). Milk protein content increased in both groups during the trial, without any group differences (*p*_period_ < 0.001). The same is true for milk protein yield (*p*_period_ < 0.001) and lactose concentration (*p*_period_ = 0.002). Milk lactose yield increased more pronouncedly in group C_60_ compared to C_35_ (*p*_C×period_ <0.001). In contrast, milk fat:protein ratio decreased in both groups, but remained more stable in group C_35_ over time (*p*_C×period_ = 0.002). (The appropriate results are shown in Table A1).

### 3.2. pH Parameters

**Figure 2:**
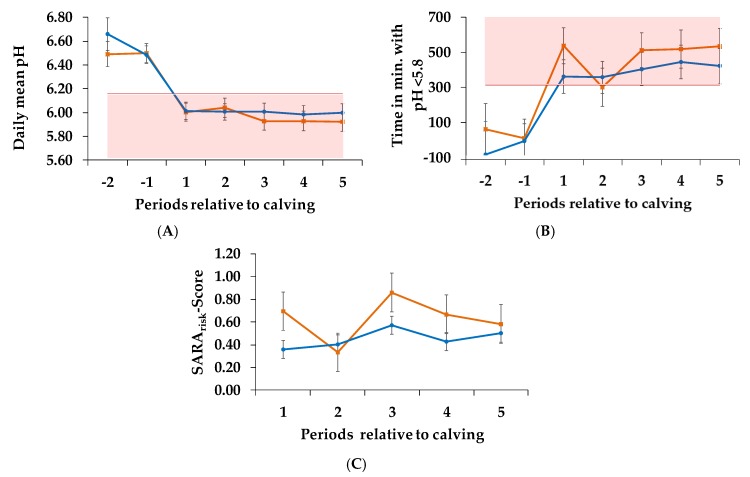
Daily mean pH of the rumen (**A**), time per day with pH < 5.8 (**B**) from week 3 antepartum until week 10 postpartum; red areas indicate SARA risk. Statistical analysis started at calving (period 1: weeks 1–2 postpartum, period 2: weeks 3–4 postpartum, period 3: weeks 5–6 postpartum, period 4: weeks 7–8 postpartum and period 5: weeks 9–10 postpartum) and SARA_risk_-Score (C) was calculated as quotient of the sum of the number of positive observations per cow in period *i* and total number of observations per cow in period *i* divided by total number of cows assessed in period *i*, during period 1–5 in the treatment groups. After calving, cows were assigned to a group with 60% concentrate feed proportion (increasing from 35 to 60% during the first three weeks after parturition, **C_60_**, **orange**, n = 6) and a group with 35% concentrate feed proportion (**C_35_**, **blue**, n = 7) in the ration.

We determined different pH parameters in order to describe the conditions in the rumen. For the SARA defining thresholds according to Zebeli et al. [9], including the daily mean pH (Figure 2A, Table 3) and the time per day with pH < 5.8 (Figure 2B, Table 3) we could not prove any significant effects between the two trial groups receiving different concentrate proportions. Regarding the SARA_risk_-Score (Figure 2C, Table 3), values for the C_60_ group ranged between 0.33 and 0.86 (±0.17 SD). The C_35_ group reached values between 0.36 and 0.57 (±0.08 SD).

**Figure 3:**
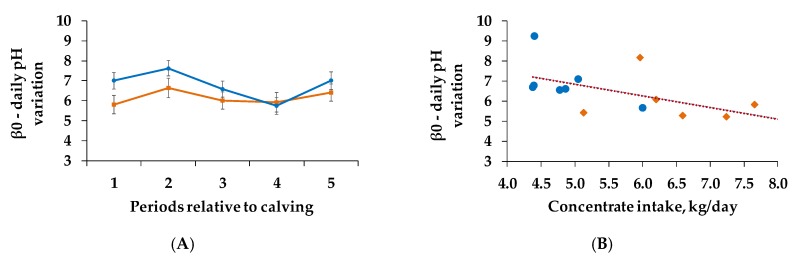
β0 (**A**: illustrates the slope of the regression line at the inflection point and therefore displays the variation of rumen pH over 24 h; the greater the values the more constant the ruminal pH) during period 1 (weeks 1–2 postpartum), period 2 (weeks 3–4 postpartum), period 3 (weeks 5–6 postpartum), period 4 (weeks 7–8 postpartum) and period 5 (weeks 9–10 postpartum) as well as regression of β0 and actual concentrate intake in period 1 (**B**: y = -0.558x + 9.589, r^2^ = 0.311, p <0.05) for each individual cow in period 1. After calving, cows were assigned to a group with 60% concentrate feed proportion (increasing from 35 to 60% during the first three weeks after parturition, **C_60_**, **orange**, n = 6) and a group with 35% concentrate feed proportion (**C_35_**, **blue**, n = 7) in the ration.

C (*p* = 0.099) tended to affect β0 (Figure 3A, Table 4), as the group with the lower C exhibited higher values and thus had lower pH variations over the day.

β0 increased during the first part of the trial to decrease during period 3 and 4, with a second peak in period 5 in both groups (*p* = 0.032). However, none of the factors affected β1 (Table 5).

**Figure 4:**
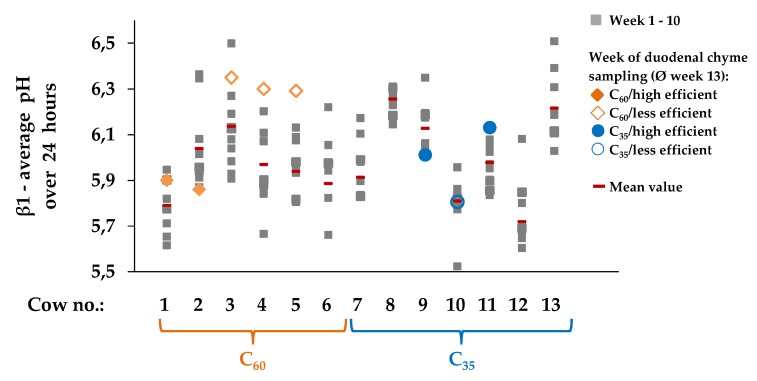
β1 (describing the inflection point of the curve and representing the average pH of the 24 h period) for individual cows and each of the 10 experimental weeks as well as of the week of duodenal chyme sampling (on average week 13 p.p. ± 16 days)) reflecting the individual variability independent of grouping. After calving cows were assigned to a group with 60% concentrate feed proportion (increasing from 35 to 60% during the first three weeks after parturition, **C_60_**, **orange**, n = 6) and a group with 35% concentrate feed proportion (**C_35_**, **blue**, n = 7) in the ration. For the week of duodenal chyme sampling, cows were additionally grouped into a **more** (●/**♦** filled, 206 ± 17 g/kg, n=5) and a **less efficient** (**○**/**◊**, blank, 122 ± 17 g/kg, n=4) group in compliance with the average microbial efficiency of 156 g/kg defined as synthesized microbial crude protein/fermented organic matter according to GfE [15].

When assessing the results of β1 on an individual basis (Figure 4) it also becomes apparent that animals within the same group differ. Both groups contain cows with lower or higher levels of average pH.

### 3.3. Short Chain Fatty Acids

Increase of total volatile fatty acids (Table 6) was observed in both groups over the trial (*p*_period_ = 0.020). Acetic acid (Table 6) tended to increase more pronounced in group C_35_ compared to C_60_ over time (*p*_C_ × *period* = 0.053). In contrast, propionic acid (Table 6) increased more pronounced in group C_60_ and decreased in group C_35_ during the last part of the trial (*p*_C_ × *period* = 0.064). Butyric acid (Table 6) decreased over time without any differences between groups (*p*_period_ = 0.001). For acetate:propionate ratio (Table 6), both groups achieved the highest values in period 1. Values then decreased during the first half of the study and increased again during the last part (*p*_period_ < 0.001).

### 3.4. Evaluations of The Week of Duodenal Chyme Sampling by Different Statistical Evaluations

#### 3.4.1. Microbial Efficiency, pH Parameters, Nutrient Flows and Digestibiliy at the Duodenum

For the analysis of the estimated parameters in the duodenal chyme samples, we wanted to assess both the effect of rations differing in C, as well as individually different microbial efficiencies. For this, different statistical evaluations were applied and cows were assigned to different C according to the initial experimental design and additionally grouped according to microbial efficiency (mCP/fOM, more and less efficient). This attempt was made in order to gain more information about the relation between pH parameters and microbial efficiency, as well as on parameter such as nutrient flows and digestibility.

#### 3.4.2. pH Parameters

We compared daily mean pH, time per day with pH < 5.8, as well as β0 and β1 during the week of duodenal sampling between the C_60_ and C_35_ groups, as well as between more and less efficient groups. However, we could not detect any significant differences (Table 7).

In this trial, concentrate had no significant effect on mCP, fOM or mCP/fOM (Table 8).

The same is true for DMF, organic matter flow (OMF) and rumen ammonia-N (Table 8).

mCP (Table 8) tended to be higher for the more efficient group compared to the less efficient group (*p*_mCP/fOM_ = 0.081). FOM (Table 8) was significantly higher for less efficient groups (*p*_mCP/fOM_ = 0.024). mCP/fOM was significantly higher for more efficient animals (*p*_mCP/fOM_ <0.001). For DMF (Table 8), as well as for duodenal organic matter flow (OMF, Table 8), we detected a significant difference between groups (*p*_mCP/fOM_ DMF = 0.032, *p*_mCP/fOM_ OMF = 0.037) as more efficient groups had higher values in both parameters.

Rumen ammonia-N (Table 8) did not differ between groups.

#### 3.4.3. Digestibility

We assessed both the total digestive tract digestibility of dry matter (tdDM) and the digestibility at the duodenum. None of the nutrient digestibilities at the duodenum (Table 9) was influenced by C.

In the same manner digestibility of ADF_om_ (Table 9) did not differ between more and less efficient animals. However, digestibility of aNDF_om_ (Table 9) tended to be higher in the less efficient group compared to the more efficient group (*p*_mCP/fOM_ = 0.086). Furthermore, digestibility of OM at the duodenum (Table 9) was significantly lower in more efficient animals (*p*_mCP/fOM_ = 0.001).

**Figure 5:**
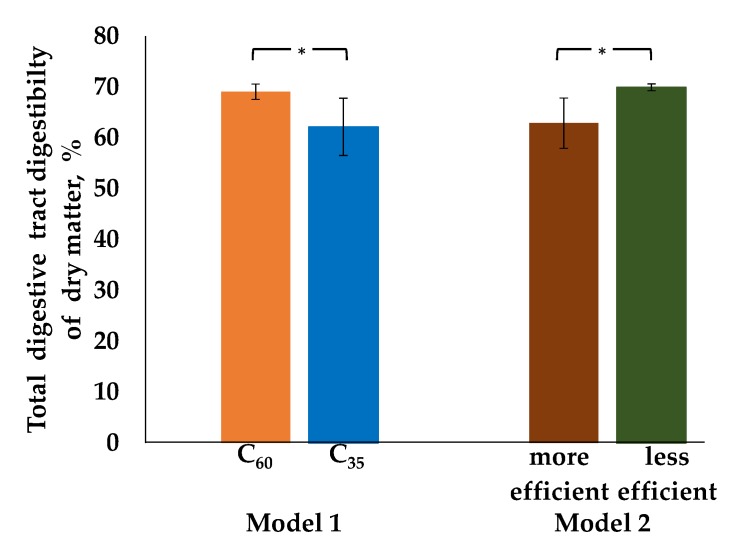
Total digestive tract digestibility of dry matter for the week of duodenal chyme sampling (on average week 13 postpartum ± 16 days). After calving cows were assigned to a group with 60% concentrate feed proportion (increasing from 35 to 60% during the first three weeks after parturition, **C_60_**, **orange**, n = 5) and a group with 35% concentrate feed proportion (**C_35_**, **blue**, n = 4) in the ration (model 1). For the week of duodenal chyme sampling, cows were additionally grouped into a **more** (206 ± 17 g/kg, **red**, n = 5) and a **less** efficient (122 ± 17 g/kg, **green**, n = 4) group in compliance with the average microbial efficiency of 156 g /kg, defined as synthesized microbial crude protein/fermented organic matter according to GfE [15] (model 2). *Indicating significant group differences. C_60_ vs. C_35_: *p*-value = 0.030, more efficient vs less efficient: *p*-value = 0.026.

The tdDM differed significantly between C_60_ and C_35_ groups (*p*_c_ = 0.030, Figure 5) as well as between more and less efficient groups (*p*_mCP/fOM_ = 0.026, Figure 5). In the first case, higher C led to higher digestibility. In the second case, less efficient cows exhibited higher values.

The same is true for the total digestive tract digestibility quotient of OM (*p*_c_ = 0.003, *p*_mCP/fOM_ = 0.027, Table 9). C_60_ and C_35_ animals did not differ in their total digestive tract digestibility quotient of aNDF_om_ and ADF_om_ (Table 9). Furthermore, the less efficient group had a significantly higher total digestive tract digestibility quotients of aNDF_om_ (*p*_mCP/fOM_ = 0.036, Table 9) and ADF_om_ (*p*_mCP/fOM_ = 0.017, Table 9) compared to the more efficient group.

### 3.5. Correlations and Regression Analysis

However, as we found a tendency of β0 being influenced by different concentrate feed proportions of the ration, we attempted to examine this relation more closely. Therefore, we correlated the β0 values with the actual concentrate intake of period 1 and then detected a significant relation (r^2^ = 0.311, *p* < 0.05, Figure 4B). Animals which consumed more concentrate feed exhibited smaller β0 values and therefore had a more fluctuating ruminal pH over the day.

#### Ruminal pH, Duodenal Nutrient Flows, and Microbial Crude Protein

We calculated further correlations and performed regression analysis with measured and calculated variables of the duodenal chyme collection to further assess the assumed pH influence on microbial efficiency, which was not verifiable in the intergroup comparison.

**Figure 6:**
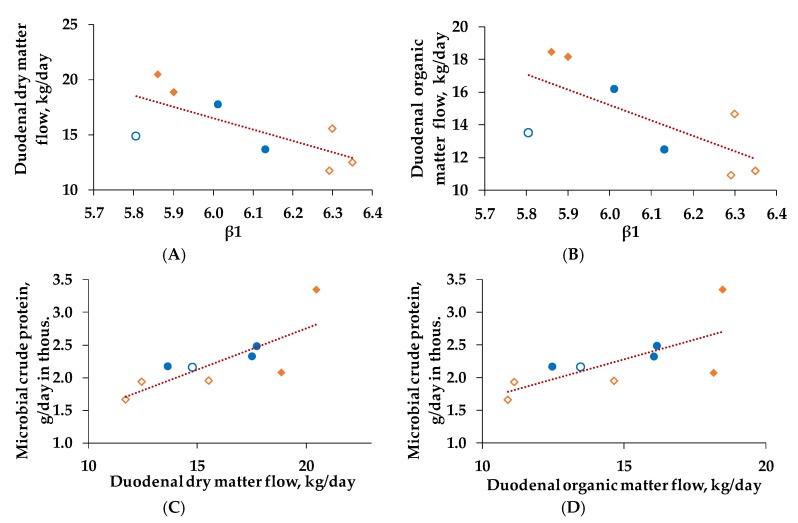
Regression of β1 (describing the inflection point of the curve and representing the average pH of the 24 h period) on duodenal dry matter flow (DMF, **A**, y = -10.25x + 77.97, r^2^ = 0.598, *p* < 0.05) and of β1 on duodenal organic matter flow (OMF, **B**, y = -9.42x + 71.74, r^2^ = 0.594, *p* < 0.05), as well as of DMF microbial crude protein (**C**, y = 0.13x + 0.23, r^2^ = 0.646, *p* < 0.05) and of OMF on microbial crude protein (**D**, y = 0.12x + 0.47, r^2^ = 0.536, *p* = 0.061) for the duodenal fistulated cows (n = 9) for the week of duodenal chyme sampling (in average week 13 postpartum ± 16 days). After calving cows were assigned to a group with 60% concentrate feed proportion (increasing from 35-60% during the first three weeks after parturition, **C_60_**, **orange**, n = 5) and a group with 35% concentrate feed proportion (**C_35_**, **blue**, n = 4) in the ration. For the week of duodenal chyme sampling cows were additionally grouped into a **more** (206 ± 17 g/kg, ●/**♦**, n = 5) and a **less efficient** (122 ± 17 g/kg, **○**/**◊**, n = 4) group in compliance with the average microbial efficiency of 156 g/kg, defined as synthesized microbial crude protein/fermented organic matter according to GfE [15]. Correlation of (**A**) and (**B**) was conducted with eight animals, due to a missing β1 value for one more efficient cow of C_35_.

Hereby, we could detect a positive relation between β1 and DMF (r^2^ = 0.598, *p* < 0.01, Figure 6A), as well as between β1 and OMF (r^2^ = 0.594, *p* < 0.01, Figure 6B) for the week of duodenal chyme collection. Furthermore, increasing DMF was associated with a higher flow of microbial crude protein (r^2^ = 0.646, *p* < 0.05, Figure 6C). The same is true for OMF (r^2^ = 0.536, *p* = 0.061, Figure 6D).

## 4. Discussion

The aim of the present study was to examine the ruminal pH caused by differing concentrate feed proportions, thereby paying special attention to diurnal pH-kinetics. The second objective of the study was to gain information on the interplay between ruminal pH parameters and microbial efficiency. For this, cows were additionally (retrospective) grouped according to their individual microbial efficiency. Therefore, the mean microbial efficiency of 156 g mCP/kg fOM according to GfE [15] was used as threshold for allocating the cows to more (>156 g/kg) or less (<156 g/kg) efficient. Moreover, efficiency related parameters, such as nutrient flows and digestibilities were examined.

In the transition period, cows decrease dry matter intake (DMI) and net energy intake (NEI) during the last days prior to calving and increase it again gradually after parturition [39]. This finding is substantiated by the significantly stronger increase in NEI and starch intake of group C_60_ resulting both from the slightly enhanced DMI and the higher NE_L_- and starch content of the ration.

We had expected that the higher starch intake of this group would lower the ruminal pH. A higher starch intake had been shown to increase the production of SCFA and thus decrease ruminal pH [5]. However, in the present study, we could not prove this inverse relation to pH parameters such as daily mean pH, time per day with pH < 5.8 and β1. Considering the SARA defining thresholds according to Zebeli et al. [9], described as a daily mean pH < 6.16 and the time per day with pH < 5.8 for more than 5.24 h, we would have expected that cows fed higher C would decrease daily mean pH and increase time with pH < 5.8 more drastically than the C_35_ group. Surprisingly both groups achieved the critical ranges defined by Zebeli et al. [9]. However, high standard errors suggest a high individual variability, which probably prevented the detection of significant differences between groups. Other studies showed the opposite by observing a decrease in ruminal pH resulting from higher concentrate intake [40,41]. Possibly, difference of starch content between the rations in the present study must have been higher to provoke extremes. On the contrary, studies of Ueda et al. [5] and Beauchemin and Penner [1] confirm our results. Ruminal pH is not only influenced by ration composition, but also by the functionality of the epithelium and thus by absorption capacity to eliminate SCFA. Neutralization by buffers and passage to lower digestive tract are considered to be further factors influencing ruminal pH for compensating an increased SCFA production [7]. Beauchemin and Penner [1] assumed that the feed depression before calving already reduces the functionality of rumen epithelium and increases the susceptibility to SARA. Consequently, the rumen mucosa would be incapable of even dealing with lower C proportions, which might already result in critical pH values. Our study supports these findings.

Gao and Oba [42] suggest that cows can be either tolerant towards, or susceptible to SARA. Other studies confirm this hypothesis by assuming a high individual variability in ruminal pH, due to individual variability in absorption capacity and adaptability of the epithelium as well as in feeding behavior [1,3,12]. Nevertheless, in the present study, cows with higher concentrate intake exhibited lower β0 values, reflecting a more fluctuating ruminal pH independent from group classification, which supports the assumption that individuality might have covered treatment effects De Veth and Kolver [19] already highlighted that a comparison of daily mean pH presupposes stable pH conditions and that the daily pH variation is at least equally relevant. Other studies endorse the view that pH variations over the day are more relevant than the daily mean pH [19,43]. Consequently, the relation between concentrate amount and pH parameters must not be neglected.

The susceptibility to SARA in early lactation is supposed to influence microbial efficiency by destabilizing the population of microorganisms [44]. Rapidly fermentable carbohydrates decrease the growth of cellulolytic bacteria and stimulate growth of lactobacilli [45]. These alterations might influence microbial efficiency to synthesize microbial protein related to fermented organic matter. Based on these assumptions, microbial efficiency might also be associated with pH variations. For a better understanding of these associations, we retrospectively grouped the cows according to their microbial efficiency irrespective of the initial diet-based grouping.

Different studies had already indicated a missing effect of C on microbial efficiency [5,18]. Our results support these findings, as we could not detect any differences between the C_60_ and the C_35_ groups in mCP, fOM and mCP/fOM, supporting the idea to re-group the cows according to their microbial efficiency, to gain more information.

We have hypothesized a direct relation between pH parameters and microbial efficiency. A low pH is considered to inhibit fiber digestion and thus decrease microbial efficiency in ruminants [13,14]. However, our results could not identify differences in pH parameters, neither between the more and less efficient groups nor between the C_60_ and C_35_ groups. The time with an unfavorable pH might have been too short to affect the cellulolytic bacteria [46]. Other studies have neither been successful to reduce celullose digestion by decreasing pH in ruminants or to find a direct relation between pH and microbial efficiency [18,47,48]. Oetzel [8] supports our findings and suggests that microbial response on a low pH or even SARA is slow and that multiple acidotic impacts are necessary to inhibit microbial activity. Additionally, concentration of cellulolytic bacteria is considered to be higher than necessary and thus this population remains as long as pH is not unfavorable for too long [49]. Nevertheless, in the present trial, negative relationships between β1 and DMF, as well as OMF were observed. Both parameters decreased with increasing β1 values. In turn, DMF and OMF were positively associated with microbial efficiency, possibly due to an accompanied decrease of predation by protozoa [50].

Oba and Allen [18] already indicated that a higher passage rate could increase microbial efficiency. A higher passage rate limits the microbial lysis as well as the use of energy for non-growth processes [18,51]. Rode et al. [52] demonstrated a higher passage rate with increasing concentrate proportions. Our study could not support these findings, neither for DMF nor for OMF. However, independent from C, more efficient cows showed higher DMF and OMF. These higher flows also explain the lower tdDM digestibility for more efficient cows in the present study [22]. Another possible explanation for lower digestibilities in the more efficient group might be that energy and substrate could not be used efficiently as digestibility increased, due to a fermentation rate that exceeded the microbial growth rate [18]. Sutton et al. [4] and Faichney et al. [53] already explained the missing relations in digestibility quotients at the duodenum and indicated that digestion in the colon partially compensates ruminal digestion in sheep. Conversely, missing differences in nutrient flows between C_60_ and C_35_ groups were accompanied by missing effects in digestibility. Only digestibility of OM analyzed at the duodenum was positively influenced by C, which is in line with the study of Yang et al. [12] and supports the idea of compensation in the lower digestive tract. The missing C effects in nutrient flows and digestibility in the present study might be due to an undersized difference of starch between the experimental rations.

Unexpectedly, the more efficient group exposed lower fOM values, in agreement with the study of Oba and Allen [18]. Energy from fOM is considered to be limiting factor for microbial efficiency [18]. That indicates that factors other than energy limited microbial efficiency additionally [17]. According to Clark et al. [17] these factors include synchronization of degradation of feed to permanently provide nutrients for microorganisms, content of nutrients and rumen conditions. Thus, a high individual variation for microbial efficiency can be assumed [17]. The second main limiting parameter for microbial crude protein synthesis is ammonia-N. In the present study, ammonia-N did neither differ between the C_60_ and C_35_ group nor between the more and less efficient group. Oba and Allen [18] could neither observe a direct relation to microbial efficiency. One appropriate explanation might be that a certain level of ammonia saturation was achieved in the present study. It was demonstrated that microbial crude protein synthesis exceeds the maximum with an amount of 5 mg/dL and does not increase further with increasing ammonia concentration [54]. For the present study, it may seem obvious that DMF and OMF mainly influenced microbial efficiency by decreasing predation by protozoa and energy spilling.

## 5. Conclusions

Higher amounts of concentrate did not affect daily mean pH or time with pH < 5.8. It may be assumed that individual differences among cows in ruminal pH impeded detection of significant group differences, due to high standard errors. However, we could prove a positive relationship between concentrate intake and β0 values, reflecting larger pH fluctuations. It seems that the ability to smoothly adapt to the rapid drop in pH decreases with increasing concentrate feed intake, whereby individual differences to cope with this challenge become more obvious.

Comparing the cows grouped by microbial efficiency did not reveal differences in pH parameters. However, a relation between daily pH fluctuation and DMF as well as OMF was found. DMF and OMF in turn, were positively associated with microbial protein synthesis. Consequently, microbial efficiency was at least indirectly influenced by daily pH variation.

Further research is necessary to complete and improve the understanding of the ruminal processes. Important influencing factors, such as feeding behavior, comprising meal sizes and number of meals per day, rumen functionality, including absorption capacity of the rumen epithelium, as well as saliva production and its buffering capacity and furthermore the microbiome itself were not assessed in the present study, but should also be considered.

## Figures and Tables

**Table 1:** Composition of concentrates during the dry and the lactating period.

Components, g/kg of Fresh Matter	Concentrates		
	Dry Period	Lactating Period	
		C_35_	C_60_
Soybean meal	115		
Rapeseed meal	150	400	200
Wheat	330	150	213
Barley		144	213
Maize		200	290
Dried sugar beet pulp	296	50	50
Urea	30	8	
Calcium carbonate	24	13	12
Soybean oil	15	10	10
Vitamin-mineral premix ^+^	40		
Vitamin-mineral premix ^#^		25	12

^+^ Mineral feed for dry cows, ingredients per kg according to the manufacturer’s specification: 10 g Ca; 120 g Na; 60 g P; 60 g Mg; 6 g Zn; 4 g Mn; 1.25 g Cu; 100 mg I; 50 mg Se; 35 mg Co; 800,000 IU vitamin A; 100,000 vitamin D_3_; 1500 mg vitamin E. ^#^ Mineral feed for lactating dairy cows, ingredients per kg according to the manufacturer’s specifications: 140 g Ca; 120 g Na; 70 g P; 40 g Mg; 6 g Zn; 5.4 g Mn; 1 g Cu, 100 mg I; 40 mg Se; 25 mg Co; 1,000,000 IU vitamin A; 100,000 IU vitamin D_3_; 1500 mg vitamin E.

**Table 2:** Chemical components of the dry period diet, as well as of the C_35_- and C_60_-ration during the experimental period from day 21 antepartum until day 70 postpartum.

Chemical Composition	Dry Period Diet	C_35_-Ration	C_60_-Ration
Dry matter, g/kg	239	251	198
Nutrients, g/kg DM ^§^			
Crude ash	63	96	59
Crude protein	131	144	153
Ether extract	34	38	42
a ^†^ Neutral detergent fiber_om_^$^	327	353	294
Acid detergent fiber_om_^$^	225	204	169
Starch content	247	285	353
Energy ^‡^, MJ/kg of DM			
ME	10.9	11.3	11.8
NE_L_	6.6	6.9	7.3

^‡^ Calculation based on equations of GfE [16]. ^§^ Dry matter. ^†^ Assayed with a heat-stable amylase for maize silage and concentrates. ^$^ Expressed exclusive of residual ash. C_35_ group comprised seven ruminally cannulated cows, including four duodenally cannulated animals with an average parity of 3.8 (±1.5). The C_60_ group contained six ruminally and five additionally duodenally cannulated animals with an average parity of 3.4 (±1.4).

**Table 3:** *p*-values of effects of concentrate proportion of the diet (C), period and the interaction between them on daily mean pH and time in minutes with pH < 5.8.

	*p*-Values		
	C	Period ^§^	C × Period
(A) Daily mean pH	0.700	0.506	0.724
(A) Time in min. with pH < 5.8	0.557	0.205	0.545

After calving, cows were assigned to a group with 60% concentrate feed proportion (increasing from 35 to 60% during the first three weeks after parturition, **C_60_**, n = 6) and a group with 35% concentrate feed proportion (**C_35_**, n = 7) in the ration. ^§^ Period 1: weeks 1–2 postpartum, period 2: weeks 3–4 postpartum, period 3: weeks 5–6 postpartum, period 4: weeks 7–8 postpartum and period 5: weeks 9–10 postpartum.

**Table 4:** *p*-values of effects of concentrate proportion of the diet (C), period and the interaction between them on daily pH variation (β0).

	*p*-Values		
	C	Period ^§^	C × Period
(A) β0 ^$^	0.099	0.032	0.517

^$^ β0 illustrates the slope of the regression line at the inflection point and therefore displays the variation of rumen pH over 24 h; the greater the values the more constant the ruminal pH. After calving, cows were assigned to a group with 60% concentrate feed proportion (increasing from 35 to 60% during the first three weeks after parturition, **C_60_**, n = 6) and a group with 35% concentrate feed proportion (**C_35_**, n = 7) in the ration. ^§^ Period 1: weeks 1–2 postpartum, period 2: weeks 3–4 postpartum, period 3: weeks 5–6 postpartum, period 4: weeks 7–8 postpartum and period 5: weeks 9–10 postpartum.

**Table 5:** Effects of concentrate feed proportion in the diet (C) and of period β1 (describes the inflection point of the curve and represents the average pH of the 24 h period) during period 1 (weeks 1–2 postpartum), period 2 (weeks 3–4 postpartum), period 3 (weeks 5–6 postpartum), period 4 (weeks 7–8 postpartum) and period 5 (weeks 9–10 postpartum) comparing the treatment groups.

Item	Grouping ^§^		SEM ^#^	*p*-Value		
	C_60_ n = 6	C_35_ n = 7		C	Period	C × Period
β1						
Period 1	5.93	6.02	0.08	0.654	0.306	0.668
Period 2	6.07	6.02				
Period 3	5.96	6.01				
Period 4	5.92	5.98				
Period 5	5.94	5.99				

^§^ After calving cows were assigned to a group with 60% concentrate feed proportion (increasing from 35 to 60% during the first three weeks after parturition, **C_60_**) and a group with 35% concentrate feed proportion (**C_35_**) in the ration. Values are presented as least square means. ^#^ Pooled standard error of means.

**Table 6:** Effects of concentrate feed proportion in the diet (C) and period on short chain fatty acids, as well as on acetate:propionate ratio and ammonia-nitrogen (ammonia-N) concentration during period 1 (weeks 1–2 postpartum), period 2 (weeks 3–4 postpartum), period 3 (weeks 5–6 postpartum), period 4 (weeks 7–8 postpartum) and period 5 (weeks 9–10 postpartum) in the treatment groups.

Item	Grouping ^§^		SEM ^#^	*p*-Value		
	C_60_ n = 6	C_35_ n = 7		C	Period	C × period
Total short chain fatty acids, mmol/L						
Period 1	76.1	71.1	5.7	0.883	0.020	0.107
Period 2	79.7	73.5				
Period 3	72.9	90.3				
Period 4	94.8	83.5				
Period 5	79.5	80.7				
Acetic acid, Mol%						
Period 1	56.9	57.9	1.8	0.117	0.001	0.053
Period 2	51.5	56.6				
Period 3	53.4	54.1				
Period 4	50.6	57.3				
Period 5	53.4	56.3				
Propionic acid, Mol%						
Period 1	23.7	22.9	1.7	0.053	<0.001	0.064
Period 2	29.5	25.3				
Period 3	29.2	27.3				
Period 4	32.6	25.5				
Period 5	31.3	25.9				
Butyric acid, Mol%						
Period 1	14.7	14.9	0.9	0.224	0.001	0.422
Period 2	13.5	13.3				
Period 3	12.1	13.8				
Period 4	11.9	13.0				
Period 5	10.9	13.1				
Acetate:propionate ratio						
Period 1	2.49	2.63	0.22	0.103	<0.001	0.131
Period 2	1.82	2.33				
Period 3	1.90	2.04				
Period 4	1.57	2.34				
Period 5	1.73	2.30				

^§^ After calving cows were assigned to a group with 60% concentrate feed proportion (increasing from 35 to 60% during the first three weeks after parturition, **C_60_**) and a group with 35% concentrate feed proportion (**C_35_**) in the ration. Values are presented as least square means. ^#^ Pooled standard error of means.

**Table 7:** Effects of concentrate feed proportion in the ration (C) and microbial efficiency (synthesized microbial crude protein/fermented organic matter) on ruminal pH parameters.

Item ^+^	Grouping Model 1 ^$^		SD ^#^	*p*-Value	Grouping Model 2 ^§^		SD ^#^	*p*-Value
	C_60_ n = 5	C_35_ n = 4			More Efficient n = 5	Less Efficient n = 4		
Daily mean pH	6.01	6.08	0.20	0.674	6.00	6.06	0.21	0.700
Time in min. with pH < 5.8	445	266	250	0.400	395	361	288	0.873
β0 ^*^	6.09	5.54	0.87	0.391	5.84	5.93	0.85	0.893
β1 ^†^	6.14	5.98	0.20	0.359	5.98	6.19	0.19	0.186

^+^ Intergroup comparison for the week of duodenal chyme sampling (on average week 13 postpartum ± 16 days) - for both assignments (**C_60_** vs. **C_35_**, **more** vs. **less efficient**, respectively). ^$^ After calving cows were assigned to a group with 60% (increasing from 35 to 60% during the first three weeks after parturition, **C_60_**) in the ration and a group with 35% concentrate feed proportion (**C_35_**). ^§^ For the week of duodenal chyme sampling cows were additionally grouped into a **more** (206 ± 17 g/kg, n = 5) and a **less efficient** (122 ± 17 g/kg, n = 4) group according to the mean microbial efficiency of 156 g/kg, defined as synthesized microbial crude protein/fermented organic matter in compliance with GfE [15]. ^*^ illustrates the slope of the regression line at the inflection point and therefore displays the variation of rumen pH over 24 h; the greater the values the more constant the ruminal pH.^ †^ describes the inflection point of the curve and represents the average pH of the 24 h period. Values are presented as means, ^#^ Pooled standard deviation.

**Table 8:** Effects of concentrate feed proportion in the ration (C) and microbial efficiency (synthesized microbial crude protein/fermented organic matter) on synthesized microbial crude protein (mCP), fermented organic matter (fOM) and microbial efficiency (mCP/fOM), and on duodenal nutrient flows and nitrogen sources, and on ruminal nitrogen balance (RNB).

Item ^+^	Grouping Model 1 ^$^		SD ^#^	*p*-Value	Grouping Model 2 ^§^		SD ^#^	*p*-Value
	C_60_ n = 5	C_35_ n = 4			More Efficient n = 5	Less Efficient n = 4		
Microbial crude protein, g/day	2129	2280	409	0.806	2479	1922	356	0.081
Fermented organic matter, kg/day	14.4	12.9	3.0	0.457	12.1 ^B^	15.8 ^A^	2.1	0.034
Microbial crude protein/fermented organic matter, g/kg	155	186	47	0.355	206 ^A^	122 ^B^	17	<0.001
Duodenal dry matter flow, kg/day	15.8	15.9	2.9	0.954	17.7 ^A^	13.6 ^B^	2.2	0.032
Duodenal organic matter flow, kg/day	13.6	13.5	2.6	0.956	15.1 ^A^	11.7 ^B^	2.0	0.037
Rumen ammonia-N ^*^, mg/100 g	3.0	7.5	4.0	0.176	5.12	5.44	3.95	0.932

^+^ Intergroup comparison for the week of duodenal chyme sampling (on average week 13 postpartum ± 16 days) - for both assignments (**C_60_** vs. **C_35_**, **more** vs. **less efficient**, respectively). ^AB^ least square means with different superscripts differ within row. ^$^ After calving cows were assigned to a group with 60% (increasing from 35–60% during the first three weeks after parturition, **C_60_**) in the ration and a group with 35% concentrate feed proportion (**C_35_**). ^§^ For the week of duodenal chyme sampling cows were additionally grouped into a **more** (206 ± 17 g/kg, n = 5) and a **less efficient** (122 ± 17 g/kg, n = 4) group according to the mean microbial efficiency of 156 g/kg, defined as synthesized microbial crude protein/fermented organic matter in compliance with GfE [15]. ^*^ Nitrogen. Values are presented as means, ^#^ Pooled standard deviation.

**Table 9:** Effect of concentrate feed proportion of the ration and microbial efficiency (synthesized microbial crude protein/fermented organic matter) on apparent nutrient digestibility at the duodenum and the total digestive tract digestibility at faecal level.

Item ^+^	Grouping Model 1 ^$^		SD ^#^	*p*-Value	Grouping Model 2 ^§^		SD ^#^	*p*-Value
	C_60_ n = 5	C_35_ n = 4			More Efficient n = 5	Less Efficient n = 4		
Duodenal nutrient digestibility, %								
Neutral detergent fiber	37	40	12	0.750	32	46	11	0.086
Acid detergent fiber	31	35	13	0.711	27	40	12	0.144
Organic matter	43	38	10	0.490	33 ^B^	51 ^A^	5	0.001
Total digestive tract nutrient digestibility, %								
Neutral detergent fiber	46	42	6	0.298	41 ^B^	49 ^A^	4	0.036
Acid detergent fiber	40	35	6	0.338	34 ^B^	43 ^A^	4	0.017
Organic matter	67 ^A^	60 ^B^	4	0.003	61 ^B^	68 ^A^	3	0.027

^+^ Intergroup comparison for the week of duodenal chyme sampling (on average week 13 postpartum ± 16 days), for both assignments (**C_60_** vs. **C_35_**, **more** vs. **less efficient**, respectively) ^AB^ least square means with different superscripts differ within row. ^$^ After calving cows were assigned to a group with 60% (increasing from 35–60% during the first three weeks after parturition, **C_60_**) in the ration and a group with 35% concentrate feed proportion (**C_35_**). ^§^ For the week of duodenal chyme sampling cows were additionally grouped into a **more** (206 ± 17 g/kg, n = 5) and a **less efficient** (122 ± 17 g/kg, n = 4) group according to the mean microbial efficiency of 156 g/kg, defined as synthesized microbial crude protein/fermented organic matter in compliance with GfE [15]. Values are presented as means. ^#^ Pooled standard deviation.

**Figure A1:**
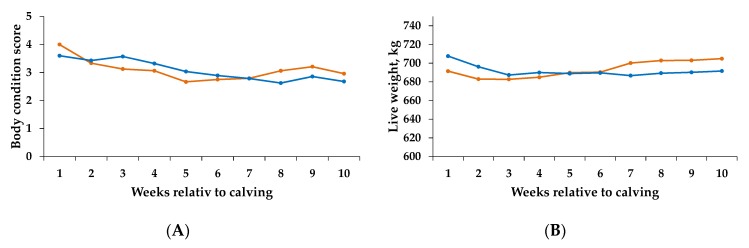
Progress of body condition score (**A**) and live weight (**B**) during the first 10 weeks postpartum. After calving cows were assigned to a group with 60% concentrate feed proportion (increasing from 35-60% during the first three weeks after parturition, **C_60_, orange**, n = 5) and a group with 35% concentrate feed proportion (**C_35_, blue**, n = 4) in the ration.

## Appendix A

**Table A1:** Effects of concentrate feed proportion in the diet (C) and period on dry matter intake (DMI), net energy intake (NEI), milk yield, energy corrected milk (ECM) and milk components during period 1 (weeks 1–2 postpartum), period 2 (weeks 3–4 postpartum), period 3 (weeks 5–6 postpartum), period 4 (weeks 7–8 postpartum) and period 5 (weeks 9–10 postpartum) in the treatment groups.

Item	Grouping ^§^		SEM ^#^	*p*-Value		
	C_60_ n = 6	C_35_ n = 7		C	Period	C × Period
DMI, kg/day						
Period 1	14.9	15.9	1.2	0.298	<0.001	0.103
Period 2	20.3	19.4				
Period 3	23.3	21.0				
Period 4	24.2	21.6				
Period 5	24.5	22.7				
NEI, MJ NE_L_/day						
Period 1	106 ^c^	113 ^b^	8	0.133	<0.001	0.016
Period 2	146 _b_	135 ^a^				
Period 3	169 ^a^	146 ^a^				
Period 4	176 ^a^	150 ^a^				
Period 5	178 ^a^	158 ^a^				
Starch intake, kg/day						
Period 1	4.6 ^a^	4.5 ^a^	0.4	0.004	<0.001	<0.001
Period 2	6.8 ^b^	5.7 ^b^				
Period 3	8.3 ^Ac^	6.1 ^Bc^				
Period 4	8.9 ^Ad^	6.2 ^Bd^				
Period 5	8.8 ^Ae^	6.6 ^Be^				
Milk yield, kg/day						
Period 1	23.3 ^e^	29.0 ^b^	1.9	0.365	<0.001	<0.001
Period 2	32.8 ^d^	36.9 ^a^				
Period 3	36.8 ^cd^	40.0 ^a^				
Period 4	40.8 ^b^	40.0 ^a^				
Period 5	41.0 ^a^	40.2 ^a^				
Milk fat content, %						
Period 1	4.88 ^a^	4.82 ^a^	0.37	0.233	<0.001	<0.001
Period 2	3.93 ^b^	4.35 ^ab^				
Period 3	3.32 ^bc^	4.07 ^b^				
Period 4	2.78 ^c^	3.82 ^b^				
Period 5	2.81 ^c^	3.79 ^b^				
Milk fat yield, kg/day						
Period 1	1.19 ^c^	1.28 ^c^	0.12	0.148	0.372	0.049
Period 2	1.30 ^bc^	1.35 ^bc^				
Period 3	1.22 ^ab^	1.51 ^ab^				
Period 4	1.14 ^a^	1.53 ^a^				
Period 5	1.17 ^a^	1.50 ^a^				
Milk protein content, %						
Period 1	3.90	3.52	0.17	0.297	<0.001	0.363
Period 2	3.34	2.96				
Period 3	3.26	3.04				
Period 4	3.23	3.16				
Period 5	3.29	3.18				
Milk protein yield, kg/day						
Period 1	0.94	1.04	0.08	0.944	<0.001	0.162
Period 2	1.11	1.09				
Period 3	1.20	1.22				
Period 4	1.33	1.27				
Period 5	1.36	1.27				
Milk lactose content, %						
Period 1	4.49	4.26	0.21	0.560	0.002	0.312
Period 2	4.74	4.31				
Period 3	4.76	4.61				
Period 4	4.79	4.80				
Period 5	4.80	4.85				
Milk lactose yield, kg/day						
Period 1	1.10 ^c^	1.23 ^b^	0.17	0.762	<0.001	<0.001
Period 2	1.57 ^b^	1.59 ^a^				
Period 3	1.75 ^b^	1.71 ^a^				
Period 4	1.97 ^a^	1.72 ^a^				
Period 5	1.97 ^a^	1.73 ^a^				
Milk fat:protein ratio						
Period 1	1.26 ^a^	1.31 ^a^	0.12	0.175	<0.001	0.002
Period 2	1.18 ^ab^	1.34 ^a^				
Period 3	1.03 ^bc^	1.29 ^a^				
Period 4	0.86 ^c^	1.21 ^a^				
Period 5	0.86 ^c^	1.19 ^a^				
ECM, kg/day						
Period 1	27.69	34.61	2.17	0.093	<0.001	0.210
Period 2	32.85	38.77				
Period 3	33.62	39.80				
Period 4	34.87	38.93				
Period 5	35.36	38.64				

^abcde^ least square means (LSMeans) with different superscripts differ within columns. ^AB^ LSMeans with different superscripts differ within row. ^§^ After calving cows were assigned to a group with 60% concentrate feed proportion (increasing from 35–60% during the first three weeks after parturition, **C_60_**) and a group with 35% concentrate feed proportion (**C_35_**) in the ration. Values are presented as LSMeans. ^#^ Pooled standard error of means.

**Table A2:** Group allocation based on the individual microbial efficiency.

Cow Number	Group Allocation	Individual Microbial Efficiency
1	More efficient	191.86
2	More efficient	215.75
3	Less efficient	114.65
4	More efficient	198.03
5	More efficient	193.02
6	Less efficient	122.20
7	More efficient	231.77
8	Less efficient	145.98
9	Less efficient	106.92

**Table A3:** Effects of microbial efficiency (synthesized microbial crude protein/fermented organic matter) on dry matter intake (DMI), milk yield, energy corrected milk (ECM) and milk components during the week of duodenal chyme sampling.

Item ^+^	Grouping ^§^		SD ^#^	*p*-Value
	More Efficient n = 5	Less Efficient n = 4		
DMI, kg/day	23.9	25.3	2.7	0.515
Milk yield, kg/day	41.2	40.3	4.7	0.783
Milk fat content, %	3.32	3.16	0.51	0.731
Milk fat yield, kg/day	1.35	1.30	0.21	0.781
Milk protein content, %	3.31	3.26	0.20	0.711
Milk protein yield, kg/day	1.36	1.35	0.18	0.923
Milk lactose content, %	4.77	4.88	0.08	0.100
Milk fat:protein ratio	0.99	0.97	0.15	0.864
ECM, kg/day	37.4	37.1	4.4	0.919

^+^ Intergroup comparison for the week of duodenal chyme sampling (on average week 13 postpartum ± 16 days), for the **more** and the **less efficient** groups. ^§^ For the week of duodenal chyme sampling cows were grouped into a **more** (206 ± 17 g/kg, n = 5) and a **less efficient** (122 ± 17 g/kg, n = 4) group according to the mean microbial efficiency of 156 g/kg, defined as synthesized microbial crude protein/fermented organic matter in compliance with GfE [15]. Values are presented as means. ^#^ Pooled standard deviation.
